# Antibiograms of Bacterial Cultures From Equine Neonates at a United Kingdom Hospital: 381 Samples (2018–2023)

**DOI:** 10.1111/jvim.70198

**Published:** 2025-08-13

**Authors:** Annabelle E. Graham, Victoria A. Colgate, Emily F. Floyd

**Affiliations:** ^1^ Institute of Infection, Veterinary and Ecological Sciences University of Liverpool UK; ^2^ Rossdales Equine Hospital Suffolk UK

**Keywords:** antimicrobials, culture, foal, resistance, susceptibility

## Abstract

**Background:**

Geographical specific data is required to guide empirical antimicrobial selection in equine neonates.

**Objectives:**

Evaluate antibiograms and survival in foals from a United Kingdom (UK)‐based hospital to guide antimicrobial selection.

**Animals:**

Blood and synovial fluid samples from 208 foals ≤ 30 days old admitted to Rossdales Equine Hospital from 2018 to 2023.

**Methods:**

Retrospective cohort study. Antimicrobial susceptibility was assessed by disc diffusion method. Bacterial culture and susceptibility and foal survival were recorded. The effects of the presence of positive culture or multidrug resistance (MDR) isolates on survival were evaluated using univariable mixed effects logistic regression.

**Results:**

Ninety‐one isolates were identified from 381 samples from 208 foals. Predominantly gram‐positive (75%, 68/91; 95% confidence interval [CI]: 65%–83%) isolates were identified, and *Enterococcus* (26%, 24/91; 95% CI: 18%–37%) was the most commonly isolated bacteria. MDR was identified in 21% of isolates (19/91; 95% CI: 13%–31%). *Enterococcus* was the most frequent MDR isolate (7/19). The combination of ampicillin and amikacin showed in vitro susceptibility in 90% (81/90; 95% CI: 82%–95%) of aerobic isolates. In total, 87% of foals were discharged from the hospital (180/208; 95% CI: 81%–91%). No association was identified between survival and the presence of positive culture or MDR isolates.

**Main Limitations:**

Retrospective design; missing data for prior antimicrobial treatment, reason for admission and admission variables.

**Conclusions and Clinical Importance:**

Ampicillin and amikacin are appropriate combination first‐line antimicrobial treatments in this population. Many Gram‐positive isolates were identified, most notably *Enterococcus*. Culture and susceptibility guided antimicrobial choices remain crucial, especially given the unpredictable susceptibility of *Enterococcus* and the frequency of MDR *Enterococcus* isolates identified.

**Abbreviations**
AST: antimicrobial susceptibility testing
CLSI: Clinical & Laboratory Standards Institute
EUCAST: European Committee on Antimicrobial Susceptibility Testing
MDR: multidrug resistance

## 1 Introduction

Sepsis remains an important cause of mortality in neonatal foals, and bacterial culture is the only method available to identify a causative agent [1]. Improved outcomes for survival have been shown when empirically selected antimicrobial regimens include an antimicrobial to which isolated bacteria are susceptible [2]. In foals with signs of sepsis, it is rarely appropriate to wait for culture and susceptibility results before starting antimicrobial treatment, and therefore empirical selection of antimicrobials is necessary [2, 3, 4]. However, making the correct empirical antimicrobial selection can be challenging. In a recent study of United Kingdom (UK) and European veterinary surgeons, third‐ or fourth‐generation cephalosporins were commonly used for treating equine neonates [5]. This practice is despite the World Health Organization (WHO) listing these drugs as highest priority, critically important antimicrobials [6], meaning they should not be used for first line treatment. The use of third‐ or fourth‐generation cephalosporins in foals is appealing because of their broad spectrum of activity, ease of administration, and lack of adverse side effects [3]. However, evidence for whether these are still appropriate for treatment of foals in the United Kingdom is lacking. Changes have occurred recently in the bacterial profiles of foals diagnosed with sepsis, from predominantly gram‐negative infections towards increasingly frequent gram‐positive infections [7, 8, 9, 10, 11]. Evidence also exists that the susceptibility of *Enterobacteriaceae* to cephalosporins is becoming less predictable, with multidrug resistant (MDR) bacteria also reported in foals [4, 7, 8, 11]. To further complicate appropriate empirical antimicrobial selection, geographical differences exist in bacterial antibiograms, which should be considered when making empirical antimicrobial selections [4, 7, 11, 12]. To our knowledge, no studies have evaluated antibiograms in foals in the United Kingdom, and current guidelines are extrapolated from studies in other countries [3]. Given the importance of protecting highest priority critically important antimicrobials and developing trends in antimicrobial resistance, selection of appropriate antimicrobials is crucial for clinical outcomes as well as minimizing antimicrobial resistance. However, blood culture has poor sensitivity, with low positive culture rates on blood cultures from foals with concurrent signs of sepsis (23.4%–49% of cultures yielding positive results) [1, 9, 10, 11, 12, 13, 14, 15, 16, 17, 18, 19, 20, 21].

The aim of our retrospective cohort study was to evaluate antibiograms from foals admitted to a UK‐based hospital. Specific study objectives were to: (a) determine the prevalence of different bacterial species isolated from blood and synovial fluid samples collected from hospitalized foals ≤ 30 days of age at Rossdales Equine Hospital and submitted to Rossdales Laboratories over a 6‐year period; (b) document antimicrobial susceptibility results to guide rational empirical antimicrobial choices for practitioners; and (c) determine if mortality was associated with positive culture or growth of MDR isolates from one or more samples.

## 2 Materials and Methods

### 2.1 Study Design and Case Selection

A retrospective review of laboratory records was performed to identify all samples submitted to Rossdales Laboratories for culture and susceptibility testing between 2018 and 2023. The following information was collected from Rossdales Equine Hospital's medical records: age, breed, sex, and survival, defined as survival to hospital discharge, of foals that had samples submitted. To be included, blood or synovial fluid samples, or both, had to be collected from foals admitted to Rossdales Equine Hospital, and that were ≤ 30 days old at the time of sample collection. Data collected on samples included type of sample submitted, bacterial isolates detected (including aerobic and anaerobic), qualitative assessment of the extent of bacterial growth (no growth, few colonies, moderate growth, profuse growth), and antimicrobial susceptibility (sensitive, intermediate, resistant) [22]. Data for admission variables, reason for referral, and if antimicrobials had been administered before referral was not available. Samples from other sites such as IV catheters, umbilical tissue, peritoneal fluid, and tracheal wash fluid were not included. A positive culture was considered to reflect a contaminant (false positive) if only scant growth was obtained in the direct culture.

### 2.2 Sample Collection

All blood culture samples were taken in a sterile manner through an IV catheter at the time of placement, or from a sterile blood collection (where the site was aseptically prepared before venipuncture). The first time collection was performed, the sample was referred to as “first blood culture.” If sequential blood cultures were taken (referred to as second, third, fourth blood culture), they were collected via sterile blood collection 24–48 h apart. All blood samples (5–10 mL) were inoculated immediately at the time of collection into a fluid culture medium bottle (2018–2022 “Bloodgrow” dual culture MW 900, Medical Wire & Equipment Co. Ltd.; from 2023 BD BACTEC Plus Aerobic [aerobic only], BD). Synovial fluid was collected aseptically and placed into a fluid culture medium bottle (2018–2022 “Bloodgrow” dual culture MW900, Medical Wire & Equipment co Ltd.; from 2023 BD BACTEC Plus Aerobic [aerobic only], BD) either at the time of sampling, or into a sterile container before being inoculated into a fluid culture medium bottle at Rossdales Laboratories. If the sample could not be immediately submitted to Rossdales Laboratories (e.g., if collected out of hours), samples were incubated at 37°C before submission. Samples were submitted for aerobic culture with or without anaerobic culture, depending on clinician preference and availability of fluid culture medium bottles.

### 2.3 Bacterial Isolation, Identification, and Classification

Samples were incubated in fluid culture medium bottles for 24 h before plating for colony identification. Samples were inoculated and incubated using standardized techniques for both aerobic and anaerobic culture (excluding 2023 when only aerobic culture was available) [23]. If multiple bacterial colony types were detected, these were subcultured onto separate plates. Bacterial colonies were identified based on colony morphology, microscopy, and biochemical testing.

### 2.4 Antimicrobial Testing

Antimicrobial susceptibility testing (AST) was carried out on all isolates using the disk diffusion method [23]. Susceptibility testing was performed for the following routinely used antibiotics: amikacin, ampicillin, amoxicillin, azithromycin, cefotaxime, ceftiofur, clarithromycin, doxycycline, erythromycin, gentamicin, marbofloxacin, oxytetracycline, penicillin, rifampicin, and trimethoprim‐sulfonamide. Susceptibility testing also was performed for cefquinome, ceftriaxone, piperacillin and tazobactam, ticarcillin and clavulanic acid. Susceptibility also was recorded for combinations of commonly used antimicrobials including ampicillin/amikacin, ampicillin/gentamicin, penicillin/gentamicin, and penicillin/amikacin. Susceptibility for chloramphenicol, sodium fuscidate, enrofloxacin, and neomycin was not included because of their infrequent use. Susceptibility to cefquinome was tested routinely from 2018 to 2020, after which it was only included on request by the clinician. Susceptibility for ticarcillin and clavulanic acid, erythromycin, and amoxicillin was not tested after 2020.

Antimicrobial susceptibility was based on zone diameter breakpoints using guidelines established by the European Committee on Antimicrobial Susceptibility Testing (EUCAST) and Clinical & Laboratory Standards Institute (CLSI) [24, 25]. Interpretation of zone sizes on isolates was performed using the Sirscan 2000 automatic AST reader or by measuring zones using calipers. As per EUCAST 2019 guidelines, only resistant (and not intermediate) results were included when defining bacteria as MDR [22]. The MDR isolates were defined as isolates that were not susceptible to ≥ 1 antimicrobial in three or more classes [26]. Intrinsic resistance was not included when defining bacteria as MDR.

### 2.5 Data Analysis

Sample size calculations indicated that information from 209 foals was required to identify an odds ratio ≥ 2.9 when the percentage of non‐surviving foals with a negative culture was between 12% and 70%, assuming 1.35 positive cultures per negative culture, a 95% confidence interval (CI) and 80% power [1]. Data analysis was performed using Excel version 16.77.1 (Microsoft) and SPSS version 24 (IBM). Numerical data was assessed for normality using the Shapiro–Wilk test and, if found to be normally distributed, presented as mean ± SD. If the data was not normally distributed, median ± interquartile range (IQR) was used. Because it was possible for multiple samples to be collected from the same foal, an interception‐only mixed effects logistic regression, with foal as a random effect, was used to evaluate clustering. The model showed evidence of clustering (estimate, 3.12; *p* < 0.001). Therefore, relationships between the binary outcome of mortality and a positive culture or presence of a MDR isolate were analyzed using univariable mixed effects logistic regression, with foal included as a random effect in all models. Statistical significance was defined as *p* < 0.05.

## 3 Results

**FIGURE 1:**
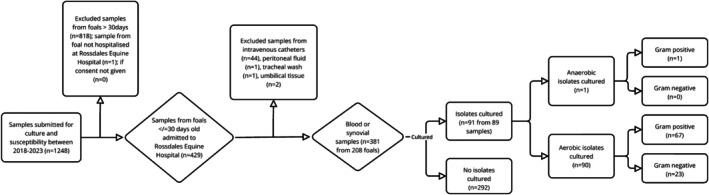
Flowchart of enrolled samples and results of bacterial culture for aerobic or anaerobic followed by Gram status.

Over the period 2018–2023, 381 samples from 208 different foals fulfilled the inclusion criteria (Figure 1). Foals were predominantly Thoroughbreds (75.9%, 158/208; 95% CI: 70%–82%), with the remaining foals predominantly Warmbloods (8.2%, 17/208; 95% CI: 5%–13%) and Irish Sports Horses (3.8%, 8/208; 95% CI: 2%–7%). Breed was not recorded in four foals. The median age of the foals at sample submission was 2 [1–7] days (median [IQR]) but varied between sample types: 2 [1–5] days for first blood culture and 19 [8–23] days for synovial samples. Samples were predominantly blood cultures (85.0%, 324/381; 95% CI: 81%–88%) and were most commonly first (50.1%, 191/381; 95% CI: 45%–55%), rather than subsequent, blood cultures. Between 2018 and 2022, when anaerobic fluid culture medium was available, 273/331 (82%) samples were tested for both aerobic and anaerobic growth.

### 3.1 Positive Culture Frequency

Only two samples grew > 1 isolate (up to two isolates) and only one sample grew an anaerobic isolate. More than one sample was submitted for 103 foals (median one sample per foal [IQR: 1–3], maximum seven samples in one foal). Positive cultures were obtained in 23.4% (89/381, 95% CI: 19%–28%) of samples and most frequently from synovial fluid (37%, 21/57; 95% CI: 24%–51%; Table 1).

**TABLE 1:** Percentage of isolates cultured from different fluid types and Gram status of those isolates from 381 samples from 208 foals.

Type of fluid sample	Number of isolates/number of samples (percentage, 95% CI)	Number of Gram‐positive isolates/total number isolates (percentage, 95% CI)	Number of Gram‐negative isolates/total number isolates (percentage, 95% CI)
First blood culture	53/191 (28%, 22%–35%)	43/53 (81%, 68%–91%)	10/53 (19%, 9%–32%)
Second blood culture	11/82 (13%, 7%–23%)	9/11 (82%, 48%–98%)	2/11 (18%, 2%–52%)
Third blood culture	4/47 (9%, 2%–20%)	3/4 (75%, 19%–99%)	1/4 (25%, 1%–81%)
Fourth blood culture	2/4 (50%, 7%–93%)	2/2 (100%, 16%–100%)	0/2 (0%, 0%–84%)
All blood cultures	70/324 (22%, 17%–26%)	57/70 (81%, 70%–90%)	13/70 (19%, 10%–30%)
Synovial fluid	21/57 (37%, 24%–51%)	11/21 (52%, 30%–74%)	10/21 (48%, 26%–70%)

### 3.2 Isolate Frequency

**FIGURE 2:**
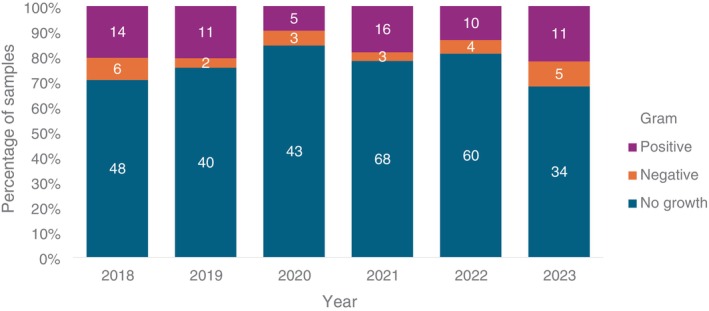
Bar chart showing the proportion of samples tested in each year of the study that yielded Gram‐positive or Gram‐negative isolates or without bacterial growth identified in each year. The numbers reported within each column are the absolute number of samples.

Of the 91 isolates identified, 75% were Gram‐positive bacteria (68/91; 95% CI: 65%–83%), and 25% (23/91; 95% CI: 17%–35%) were Gram‐negative bacteria (Figure 2). Gram status varied between fluid sample types (Table 1). The most commonly isolated bacteria were *Enterococcus*, followed by *Staphylococcus* and *Streptococcus* (Table 2). Ten foals had blood and synovial fluid sampled concurrently at admission: three foals had no growth from either sample; two foals had profuse growth of the same bacteria from each fluid source (*Klebsiella*; *Streptococcus*); one foal had profuse growth of *Streptococcus* from blood culture and profuse growth of *Actinobacillus* from a synovial sample; three foals had single positive growth from synovial fluid and not from blood culture (*Actinobacillus*; *Enterobacter*; *Streptococcus*); and one foal grew *Escherichia* on blood culture but had no growth from synovial fluid.

**TABLE 2:** Percentage of bacterial isolates detected, and types of fluid sample submitted from 381 samples from 208 foals.

Bacteria isolated	Number of isolate/total number of isolates (and percentage, 95% CI)	Fluid submission type
*Enterococcus*	24/91 (26%, 18%–37%)	Blood (*n* = 18), synovial (*n* = 6)
*Staphylococcus*	18/91 (20%, 12%–30%)	blood (*n* = 17), synovial (*n* = 1)
*Streptococcus*	18/91 (20%, 12%–30%)	Blood (*n* = 14), synovial (*n* = 4)
*Escherichia*	12/91 (13%, 7%–22%)	Blood (*n* = 9), synovial (*n* = 3)
*Bacillus*	7/91 (8%, 3%–15%)	Blood (*n* = 7)
*Klebsiella*	5/91 (5%, 2%–12%)	Blood (*n* = 3), synovial (*n* = 2)
*Actinobacillus*	4/91 (4%, 1%–11%)	Synovial (*n* = 4)
*Enterobacter*	1/91 (1%, 0.03%–6%)	Synovial (*n* = 1)
*Pasteurella*	1/91 (1%, 0.03%–6%)	Blood (*n* = 1)
*Clostridium*	1/91 (1%, 0.03%–6%)	Blood (*n* = 1)

### 3.3 Antimicrobial Susceptibility

Susceptibility results were available for all samples, but antimicrobials tested varied among samples based on disc availability and clinician preference. A maximum of 21 antimicrobials were tested against any single isolate, with a median of 15 (IQR: 15–20; minimum, 13) antimicrobials per isolate. Antibiograms were created for all aerobic, Gram‐negative and Gram‐positive bacteria as well as isolates that were identified in > 10% of all samples (Tables 3 and 4).

**TABLE 3:** Antibiograms for isolates cultured for individual antimicrobials for foals < 30 days from blood and synovial fluid. *Enterococcus*, *Staphylococcus*, *Streptococcus*, and *Escherichia* were the most commonly identified isolates.

Bacteria isolated from sample	Aminoglycosides		Ansamycin	Penicillins			Cephalosporins				Anti‐pseudo penicillin + B‐lactamase inhibitors		Fluoroquinolones	Folate pathway inhibitor	Macrolides			Tetracyclines	
	Amikacin	Gentamicin	Rifampicin	Amoxicillin	Ampicillin	Penicillin	Cefotaxime	Cefquinome	Ceftiofur	Ceftriaxone	Piperacillin and tazobactam	Ticarcillin and clavulanic acid	Marbofloxacin	Trimethoprim sulphonamide	Azithromycin	Clarithromycin	Erythromycin	Doxycycline	Oxytetracycline
All aerobes	45/85	54/90	57/90	31/36	61/87	36/90	73/74	31/36	76/90	72/87	81/88	31/33	72/90	51/90	64/87	63/88	21/33	71/90	51/89
	53%	60%	63%	86%	70%	40%	99%	86%	84%	83%	92%	94%	80%	57%	74%	72%	64%	79%	57%
Gram positive	26/62	39/67	55/67	25/26	52/64	36/67	53/54	21/25	55/67	52/65	60/66	23/24	50/67	44/67	54/66	59/66	19/24	56/67	44/66
	42%	58%	82%	96%	81%	54%	98%	84%	82%	80%	91%	96%	75%	66%	82%	89%	79%	84%	67%
Gram negative	19/23	15/23	2/23	6/10	9/23	0/23	20/20	10/11	21/23	20/22	21/22	8/9	22/23	7/23	10/21	4/22	2/9	15/23	7/23
	83%	65%	9%	60%	39%	0%	100%	91%	91%	91%	95%	89%	96%	30%	48%	18%	22%	65%	30%
*Enterococcus*	3/24	7/24	15/24	9/10	20/24	6/24	12/12	5/9	14/24	12/23	17/23	7/8	11/24	9/24	15/23	20/23	6/8	15/24	11/24
	13%	29%	63%	90%	83%	25%	100%	56%	58%	52%	74%	88%	46%	38%	65%	87%	75%	63%	46%
*Staphylococcus*	13/14	18/18	18/18	9/9	13/18	12/18	18/18	9/9	18/18	17/17	18/18	9/9	17/18	16/18	16/18	17/18	7/9	17/18	15/18
	93%	100%	100%	100%	72%	67%	100%	100%	100%	100%	100%	100%	94%	89%	89%	94%	78%	94%	83%
*Streptococcus*	4/18	7/18	18/18	4/4	15/15	16/18	18/18	4/4	18/18	18/18	18/18	4/4	15/18	12/18	17/18	17/18	4/4	17/18	13/17
	22%	39%	100%	100%	100%	89%	100%	100%	100%	100%	100%	100%	83%	67%	94%	94%	100%	94%	76%
*Escherichia*	12/12	10/12	0/12	4/8	4/12	0/12	11/11	8/9	12/12	11/11	11/11	6/7	12/12	4/12	3/10	1/11	0/7	6/12	3/12
	100%	83%	0%	50%	33%	0%	100%	89%	100%	100%	100%	86%	100%	33%	30%	9%	0%	50%	25%

**TABLE 4:** Antibiogram for combinations of commonly used antimicrobials. Number sensitive/total number tested and percentage (and 95% CI). *Enterococcus*, *Staphylococcus, Streptococcus*, and *Escherichia* were the most commonly identified isolates.

Isolate(s)	Ampicillin + amikacin	Penicillin + amikacin	Penicillin + gentamicin	Ampicillin + gentamicin
All aerobes	81/90	65/90	66/90	79/90
	90% (82%–95%)	72% (62%–81%)	73% (63%–82%)	88% (79%–94%)
Gram positive	59/67	46/67	51/67	60/67
	88% (78%–95%)	69% (56%–80%)	76% (64%–86%)	90% (80%–96%)
Gram negative	22/23	19/23	15/23	19/23
	96% (78%–100%)	83% (61%–95%)	65% (43%–84%)	83% (61%–95%)
*Enterococcus*	20/24	7/24	9/24	20/24
	83% (63%–95%)	29% (13%–51%)	38% (19%–59%)	83% (63%–95%)
*Staphylococcus*	15/18	17/18	18/18	18/18
	83% (59%–96%)	94% (73%–99%)	100% (82%–100%)	100% (82%–100%)
*Streptococcus*	15/18	17/19	16/18	15/18
	83% (59%–96%)	89% (67%–99%)	89% (65%–99%)	83% (59%–96%)
*Escherichia*	12/12	12/12	10/12	10/12
	100% (74%–100%)	100% (74%–100%)	83% (52%–98%)	83% (52%–98%)

Of the 91 isolates, 21% (19/91; 95% CI: 13%–31%) were classified as MDR from 18 foals (9%, 18/208; 95% CI: 5%–13%). One foal had an MDR *Escherichia* identified on both the first and third blood cultures. The MDR bacteria were most commonly identified from first blood cultures (9/19; 47%; 95% CI: 24%–71%). *Enterococcus* was the most common isolate that was MDR (7/19, 37%; 95% CI: 16%–62%; Table 5).

**TABLE 5:** Number of multidrug resistant (MDR) bacterial isolates detected, and types of fluid sample submitted from 381 fluid samples from 208 foals.

MDR bacteria isolated	Number of isolate/total number of isolates (percentage and 95% CI)	Fluid submission type
*Enterococcus*	7/19 (37%, 16%–62%)	Blood (*n* = 7; first culture [*n* = 4], second culture [*n* = 1], third culture [*n* = 1], fourth culture [*n* = 1])
*Escherichia*	5/19 (26%, 9%–51%)	Blood (*n* = 4; first culture [*n* = 3], third culture [*n* = 1]), synovial (*n* = 1)
*Staphylococcus*	2/19 (11%, 1%–33%)	Blood—first culture (*n* = 2)
*Actinobacillus*	2/19 (11%, 1%–33%)	Synovial (*n* = 2)
*Bacillus*	1/19 (5%, 0.1%–26%)	Blood—fourth culture (*n* = 1)
*Klebsiella*	1/19 (5%, 0.1%–26%)	Blood—second culture (*n* = 1)
*Enterobacter*	1/19 (5%, 0.1%–26%)	Synovial (*n* = 1)

### 3.4 Survival of Foals

Survival data was available for all foals and 86.5% (180/208; 95% CI: 81%–91%) survived to discharge from hospital. Of those that survived to discharge, 43.9% (79/180; 95% CI: 37%–52%) had at least one bacterial isolate cultured from at least one fluid sample, and 8.9% (16/180; 95% CI: 5%–14%) of foals had an MDR isolate cultured on any given sample. Of the 13.5% of foals (28/208; 95% CI: 9%–19%) that died or were euthanized, 36% (10/28; 95% CI: 19%–56%) had at least one bacterial isolate cultured from at least one fluid sample, and 7% (2/28; 95% CI: 1%–24%) foals had an MDR isolate cultured on any given sample. No significant associations were identified on univariable mixed effects logistic regression between mortality and the presence of an MDR isolate or mortality and a positive culture (Table 6).

**TABLE 6:** Univariable generalized mixed effect logistic regression model of risk factors associated with mortality in foals admitted to a hospital with blood or synovial samples or both submitted for culture and susceptibility.

Variable	Odds ratio	Lower 95% CI	Upper 95% CI	*p*
Positive culture	0.9	0.3	2.3	0.8
MDR identified	0.7	0.1	5.9	0.7

## 4 Discussion

### 4.1 Positive Culture Frequency

A positive culture was obtained in 23.4% of the samples obtained in our study. This result is at the lower end of what is reported in other studies (23.4%–49%) [11, 27]. This difference may be related to prior antimicrobial administration, which was not recorded in our study. However, conflicting evidence exists in foals regarding prior antimicrobial administration and lower positive culture frequency [1, 16, 18, 19]. In people, prior antimicrobial treatment can decrease positive blood culture by > 50% when given 2–72 h before blood culture, with the largest decrease (up to 9.1%) seen in the first 5 h [28]. The percentage of patients with a positive culture before IV antimicrobials, however, was only 25% [28]. Another hypothesis for lower positive culture rates was that fewer cases had sepsis. Reasons for admission and taking bacterial cultures, and variables at admission were not recorded in our study, and thus it is not possible to determine if signs of sepsis were present in these neonates [9]. It is also possible that bacterial culture techniques resulted in false negative results and therefore a lower positive culture frequency. In one study, blood culture failed to detect isolates in 40% of septic foals [29]. Currently, no standardized technique exists for collection of blood culture samples from foals. In our study, blood culture medium bottles were filled with 5–10 mL blood as previously described [11, 13, 15, 17, 18, 19]. Evidence in adult humans indicates that repeated sampling and increasing the volume from 10 to 30 mL increased the likelihood of a positive culture yield by 61% [30]. A potentially interesting observation was that a higher proportion (47%, 16/34) of samples was positive on culture in 2023 when a different type of culture medium bottle with resins was used. Evidence indicates that using resins in blood culture medium binds antimicrobials and can increase the frequency of positive culture rates of *Escherichia coli* in horses [31]. Further research is needed to determine if increasing the volume of blood collected and the type of culture medium bottle used increases the sensitivity of blood culture in bacteremic foals.

### 4.2 Isolate Frequency

The frequency of Gram‐positive isolates (75%) follows and exceeds current trends towards predominantly Gram‐positive infections in foals [7, 8, 10, 11, 17, 19, 20]. A previous study also identified 75% gram‐positive isolates, but that study only examined blood culture results [19]. The location of sampling potentially can affect the detection of gram‐negative bacteria, with an under‐diagnosis of gram‐negative infections when using blood culture alone [29]. Synovial samples in our study had a higher frequency of Gram‐negative (48%) isolates compared with blood cultures. Both Gram‐positive and Gram‐negative bacteria are identified in sepsis arising from synovial infections in horses, with a reported increasing predominance of gram‐positive isolates since 2008 [32, 33]. Only 2/10 foals that had concurrent blood and synovial samples taken had the same isolate identified on both samples. These results further emphasize the importance of sampling location, especially if localizing signs of infection are present.

Anaerobic bacteria only were identified in one sample, compared to reports of 1.2%–6% [9, 13, 15, 17, 18, 29, 33, 34, 35] with one study reporting as high as 13% [10]. Although the difference in our study could be attributed to the lack of anaerobic samples tested in 2023, 82% of the other 331 samples were tested, and therefore this factor is unlikely to have been relevant. A consensus is lacking in humans and equids regarding the routine use of both aerobic and anaerobic (dual) cultures [27, 36]. Facultative anaerobes may only be identified in anaerobic cultures compared to aerobic, supporting the use of dual cultures in people [36, 37, 38]. Where collection volume is limited, such as in blood samples or synovial fluid samples in neonates, it may be beneficial to submit a single aerobic culture to increase the volume in a single sample and improve detection rates [27, 30]. Our results do not support an emerging trend in anaerobic culture as hypothesized previously [10]. However, more research is needed to determine if anaerobic infections affect outcome in equine neonates and therefore whether routine inclusion of anaerobic cultures is necessary given their low incidence.

*Enterococcus* was the most commonly isolated bacteria in our study. *Enterococcus* spp. was identified as the most common cause of bacteremia in 29% of foals with diarrhea in one study [16]. The indication for performing fluid culture was not recorded in our study, and thus it is possible that this finding is due to the underlying disease process or selection due to prior antimicrobial treatment. *Enterococcus* has emerged in recent years as an increasingly important cause of sepsis in humans and foals, and the high prevalence in our study is concerning [7, 11, 39]. In England, there also has been a recent trend of *Enterococcus faecium* being more frequently identified than *Enterococcus faecalis* in humans [40]. In cases of sepsis in humans, *E. faecium* has been associated with higher mortality and antimicrobial resistance compared with *E. faecalis* [40]. Two previous studies reported a higher prevalence of *E. faecium* than *E. faecalis* in foals [7, 41]. Increased antimicrobial resistance was reported previously in foals with *E. faecium* compared with *E. faecalis*, and foals that cultured *Enterococcus* were significantly less likely to survive than foals that cultured other bacteria [41]. It was not possible to determine *Enterococcus* species in our study, but given the emerging trends, ongoing surveillance of enterococcal species in horses, along with restrictive use of antimicrobial drugs, is crucial to prevent further resistance [42].

### 4.3 Antimicrobial Susceptibility

Some of the antimicrobials evaluated in our study are classified as critically important antimicrobials by the WHO [6]. These drugs should be reserved for cases in which no other alternatives are effective and only after appropriate susceptibility testing or when evidence for their use in certain diseases is compelling [43].

Our study is in agreement with other research that the combined use of ampicillin and amikacin in foals is an appropriate choice for both Gram‐negative and positive isolates [2, 10]. Ampicillin also showed good in vitro efficacy against *Enterococcus*, further supporting its use in this setting [41, 44]. Although it has been suggested, given increased resistance of *E. faecium* to ampicillin, that chloramphenicol would be a more appropriate choice [41]. Chloramphenicol was not included in our study because it was infrequently tested during the study period, and enterococcal species identification was not performed routinely. Chloramphenicol has substantial drawbacks to its use in neonates, including limited Gram‐negative enteric spectrum, bacteriostatic nature, lack of IV formulation, and risk of toxicity in humans [44]. Chloramphenicol may be an appropriate choice for older foals with localized *Enterococcus* infections, such as those of the lower urogenital tract, but its use should be guided by culture and susceptibility testing with consideration of its pharmacokinetics [41, 44]. The pharmacokinetics of chloramphenicol in equine neonates changes significantly in the first 6 weeks [45, 46]. Therefore, the dose and frequency of dosing must be appropriate for the age of the foal. Even at higher doses, the use of chloramphenicol may be limited to bacteria with a minimum inhibitory concentration (MIC) < 2 μg/L because of inadequate serum concentrations needed for therapeutic efficacy [47, 48].

Our study supports the possible use of cephalosporins for foals in which aminoglycosides are not an option (e.g., those with concurrent azotemia) [3]. Breakpoints for cephalosporins have not been established in foals, but the labeled dosage of ceftiofur for adults is considered to be effective against most Gram‐positive respiratory tract pathogens [49]. The higher dosages of ceftiofur used in neonates could provide sufficient in vivo susceptibility for bacteria with higher in vitro breakpoints, such as Gram‐negative bacteria, but are unlikely to be effective for *Enterococcus* because of intrinsic resistance [49, 50]. Cefotaxime was the most effective antimicrobial in vitro for all aerobic isolates (99%, 73/74; 95% CI: 93%–100%). However, these include *Enterococcus*, which has intrinsic resistance to cephalosporins and has unpredictable in vivo susceptibility patterns to other antimicrobials [7, 17, 41]. Consequently, susceptibility results are even more important if *Enterococcus* is identified. If *Enterococcus* is removed from the antibiogram, cefotaxime and ceftiofur are still effective in 98% (61/63) and 94% (62/66) of all aerobes, respectively. However, despite the popular use of ceftiofur for foals in the UK, because of the prevalence of *Enterococcus*, cephalosporins may not be considered a good empirical antimicrobial choice in this population [5, 44].

Other beta‐lactam antimicrobials that were effective in vitro were ticarcillin‐clavulanic acid and piperacillin‐tazobactam. The use of these antipseudomonal penicillin antimicrobials is reported infrequently in veterinary literature and both antimicrobials were classified in 2018 under WHO guidelines for “human use only” [6, 51, 52, 53].

### 4.4 MDR Isolates

Few studies have reported the frequency of MDR isolates in fluid cultures from foals, with a reported range of 13%–44.3% for all isolates [8, 10, 11, 17, 41]. In our study, *Enterococcus* was the most commonly isolated bacteria and the most likely to be MDR (29% [7/24] of all *Enterococcus* isolated were MDR). *Enterococcus* spp. have intrinsic in vivo resistance to cephalosporins, aminoglycosides, and potentiated sulfonamides, and these groups were not included when defining MDR. Exclusion of intrinsically resistant antimicrobials when reporting MDR isolates is not consistently performed across studies, making comparison among studies difficult. In one study, 59% of *Enterococcus* were MDR and associated with a higher mortality rate in foals compared to non‐MDR *Enterococcus* [41]. Too few samples were available to investigate an association in our study for MDR compared with non‐MDR *Enterococcus*. However, it is concerning that 21% of all isolates tested were MDR, further emphasizing the importance of performing susceptibility testing in foals to ensure appropriate antimicrobial selection, especially for MDR isolates [10].

### 4.5 Survival of Foals

Survival of hospitalized, critically ill neonatal foals since 2007 is reported to be 52%–80.6% [1, 9, 10, 11, 16, 17, 18, 20, 34, 35, 54]. Because sepsis scores and variables at admission were not evaluated, the higher survival rate (87%) in our study could be related to case selection bias, less severe disease, or lower sepsis scores in our population. Caution is required when comparing survival results between studies due to the differences in defining the diagnosis of sepsis and inclusion criteria. However, the proximity of referrals is an important factor that could influence survival in this population. Most referrals are within 60 min of the hospital, and decreased duration of disease before referral has been associated with better outcomes in sepsis in foals [55]. Our results are in agreement with those of other studies in that survival was not associated with the presence of a positive culture [11, 16, 17, 18, 20, 54, 55]. In agreement with other studies, foals identified with a MDR bacteria were not less likely to survive compared to foals when no MDR isolates were detected [10, 11]. We did not evaluate whether correct selection of antimicrobials impacted survival, but this factor has been discussed in other studies [2, 10].

### 4.6 Limitations

The most important limitation of our study was its retrospective design and missing data regarding admission variables, reason for referral, and whether or not antimicrobials had been administered before referral. Case selection bias was present because a single hospital was used with a mostly Thoroughbred population. This choice was made to minimize inconsistency in sampling technique and sample handling. Antibiograms vary geographically and therefore extrapolation of our results to other populations should be made cautiously [56]. Antibiograms ideally are created and used by individual hospitals or groups of hospitals because the source population will be most applicable to the patient [57]. Therefore, development of regional or local antibiograms is recommended as part of antimicrobial stewardship in veterinary medicine [56, 57, 58]. Because of the duration of the study, inconsistencies existed with the type of blood culture medium bottle and availability of discs for AST. The disc diffusion technique has inherent limitations compared with other methods such as antimicrobial gradient or broth dilution methods [59]. Disc diffusion is a well‐standardized technique with zone diameter breakpoints, but fewer veterinary breakpoints are available compared with MIC breakpoints [25]. Disc diffusion is a widely used AST method in clinical laboratories, and the results are relevant to practitioners [23]. Newer methods for faster isolate identification such as matrix‐assisted laser desorption ionization‐time‐of‐flight (MALDI‐TOF) mass spectrometry are reported in veterinary species, with some commercial laboratories in the United Kingdom starting to use this method for isolate identification [60]. However, for all methods used, it is important to remember that in vitro does not equate to in vivo susceptibility, especially when considering the in vivo synergistic effects of some antimicrobial combinations [44]. This factor should be considered when interpreting susceptibility results in clinical cases.

## 5 Conclusion

Our results support the use of ampicillin and amikacin in combination in this population of equine neonates as a first‐line antimicrobial. A high frequency of gram‐positive isolates was found, most notably *Enterococcus*. The use of culture and susceptibility to guide antimicrobial choices remains critically important. Given the unpredictable susceptibility of *Enterococcus* and frequency of MDR *Enterococcus* isolates in our study, the value of culture and susceptibility has been further emphasized to ensure appropriate antimicrobial choices.

## Disclosure

This retrospective paper discusses the susceptibility testing of amoxicillin, piperacillin and tazobactam, clarithromycin, ceftriaxone, amikacin, rifampicin, doxycycline, ampicillin, ticarcillin and clavulanic acid, erythromycin, cefotaxime, azithromycin, cefquinome, and marbofloxacin but not used in the study.

## Ethics Statement

Research ethics committee oversight not required for retrospective study of clinical records. Owners signed consent forms that explained that data from the medical records might be used for research in general. Authors declare human ethics approval was not needed.

## Conflicts of Interest

The authors declare no conflicts of interest.
